# Biological, ecological, conservation and legal information for all species and subspecies of Australian bird

**DOI:** 10.1038/sdata.2015.61

**Published:** 2015-11-10

**Authors:** Stephen T. Garnett, Daisy E. Duursma, Glenn Ehmke, Patrick-Jean Guay, Alistair Stewart, Judit K. Szabo, Michael A. Weston, Simon Bennett, Gabriel M. Crowley, David Drynan, Guy Dutson, Kate Fitzherbert, Donald C. Franklin

**Affiliations:** 1 Research Institute for the Environment and Livelihoods, Charles Darwin University, Darwin, NT 0909, Australia; 2 Department of Biological Sciences, Macquarie University, North Ryde, NSW 2109, Australia; 3 BirdLife Australia, 5/60 Leicester St, Carlton, Vic. 3053, Australia; 4 Institute for Sustainability and Innovation, Victoria University, Footscray Park Campus, PO Box 14428, Melbourne MC, Vic. 8001, Australia; 5 East Asian—Australasian Flyway Partnership Secretariat, 3F G-Tower, 175 Art center-daero (24-4 Songdo-dong), Yeonsu-gu, Incheon 406-840, Republic of Korea; 6 Centre for Integrative Ecology, School of Life and Environmental Sciences, Faculty of Science, Engineering and the Built Environment, Deakin University, 221 Burwood Hwy, Burwood, Vic. 3125, Australia; 7 28 Morgan Crescent, Curtin, ACT 2605, Australia; 8 The Cairns Institute, James Cook University, PO Box 6811, Cairns, Qld 4870, Australia; 9 Australian Bird & Bat Banding Scheme, GPO Box 8, Canberra, ACT 2601, Australia; 10 School of Life and Environmental Sciences, Deakin University, Waurn Ponds, Vic. 3216, Australia; 11 Bush Heritage Australia, PO Box 329, Flinders Lane, Melbourne, Vic. 8009, Australia

**Keywords:** Conservation biology, Zoology, Biodiversity, Taxonomy

## Abstract

We introduce a dataset of biological, ecological, conservation and legal information for every species and subspecies of Australian bird, 2056 taxa or populations in total. Version 1 contains 230 fields grouped under the following headings: Taxonomy & nomenclature, Phylogeny, Australian population status, Conservation status, Legal status, Distribution, Morphology, Habitat, Food, Behaviour, Breeding, Mobility and Climate metrics. It is envisaged that the dataset will be updated periodically with new data for existing fields and the addition of new fields. The dataset has already had, and will continue to have applications in Australian and international ornithology, especially those that require standard information for a large number of taxa.

## Background & Summary

Extensive datasets of faunal attributes play an important role in the analysis and understanding of biological systems and the conservation of biodiversity. For birds, they have been used, for example, to identify traits predictive of extinction risk^1^, to predict vulnerability to recent and impending climate change^2,3^, and to identify the correlates of invasion success^4^. Often these datasets are compiled *de novo* from primary sources for each application, leading to duplication of effort and repetition of the opportunity for error, as well as impeding replication of analyses.

To overcome these problems for Australian birds, we introduce a dataset (Data Citation 1) with associated metadata and reference list for free use by researchers and other interested persons. Ecological, biological and conservation information is provided for every species and subspecies recorded in Australia since European settlement, including vagrants and extinct taxa, with separate entries for Australian breeding and non-breeding populations of seabirds, taxa that have been officially but erroneously reported and introduced species that subsequently died out, 2,056 taxa or populations in total (Table 1). For the purpose of this database, Australia is defined to include the Australian mainland and continental islands, Australian territories (excluding the Australian Antarctic Territory) and the Australian Fishing Zone, which extends 200 nautical miles (370 km) off the coast of the above-named areas. Version 1 of the database contains 230 fields of data for each relevant taxon, grouped into 15 categories (Table 2). Of eight data types, the most frequent are binary, followed by numeric values and unranked categories other than binary (Table 3).

The Australian avifauna features high rates of endemism at species and higher taxonomic levels up to that of families, especially among the passerines. Over 60% of all terrestrial birds globally are passerines and Australia was the major early centre of their radiation^5,6^. Many Australian passerines are cooperative breeders such that the continent has a high incidence, and the highest proportional incidence, of cooperative breeding species globally^7^. Australia was also likely to have been the major centre for early diversification of parrots^6^ and remains rich in parrot and cockatoo species. Australian birds, therefore, have a role in global ornithology that is at once both unique and central.

We envisage that the dataset (Data Citation 1) will have a wide range of applications in Australian as well as international ornithology, with particular relevance to analyses which require standard information for a large number of taxa. It was initially developed in 1990 to support the first national assessment of the conservation status of all Australia’s avian species and subspecies^8^ and was then published as a 3.5 inch disk^9^. It was refined to support subsequent status assessments of extinction risk^10,11^, analyses of population trends in Australian birds^12^ and analyses of the risk and potential management of climate change^13,14^. It was built concurrently with the *Handbook of Australian, New Zealand & Antarctic Birds* (HANZAB)^15–21^ and subsequently cross-checked against it. Recent applications independent of its construction are as a source of standard body mass data for the calculation of relative brain size in 504 Australian bird species^22^, and an analysis of flight initiation (escape) distances of 250 species^23^.

We intend to provide progressive updates with expansion of its coverage. Management of the data, including oversight of taxonomy, format, eligibility and peer review of new columns, will be overseen by a committee answerable to the BirdLife Australia Research and Conservation Committee. The new version will specify any changes from previous versions. Co-authorship of future versions will be available to anyone contributing a full column of data with attribution of the data to original sources.

## Methods

Order names follow Jarvis *et al.* ^24^ and family and generic names follow Dickinson and Remsen^25^ and Dickinson and Christidis^26^, thus following the most-recent synopsis of developments in the understanding of higher-level relationships among all bird taxa. To allow seamless international comparison of trends in conservation status, species definitions are those of BirdLife International^27^ and BirdLife Australia, which are based on the use of ‘divergence between undisputed sympatric species as a yardstick for assessing the taxonomic status of allopatric forms’^28^. Subspecies taxonomy and nomenclature largely follows HANZAB^15–21^ unless this has been updated by recent research. Where this recent research includes genetic analyses, we adopt the ‘precautionary view that, while genetic studies can detect differences between populations, some of which may not readily be apparent in the phenotype, a failure to detect variation may reflect incomplete sampling of the genome’^11^.

Data were variously sourced (copied), derived from or extracted from a wide variety of sources, or interpreted *de novo*, as documented for each field in the metadata (Data Citation 1, file ‘Australian_Bird_Data_Version_1_Metadata.csv’). In many cases, a primary source that provided data on core or common or breeding or native Australian taxa^15–21^ was complemented with one or more secondary sources for taxa that are rare visitors, which do not breed in Australia, or have been introduced to it. Almost every field required some interpretation for a few taxa to align data from past with current taxonomies; this was mostly straightforward because most such changes involve elevation or reduction of geographically-discrete taxa between species and subspecies levels. However, many fields required greater levels of interpretation as discussed in the following paragraphs.

One issue is that source data were not necessarily systematic and so varied in quality between taxa. For example, source data on food types is variously quantitative, qualitative, or admixtures of these. In this case, data were converted to binary scores of ‘non-trivial’ usage for each of a number of categories of food type or feeding substrate. An analogous interpretative process of categorisation was used for many ecological fields such as feeding and breeding habitats. Where possible, such categories were sourced from external authorities, and in all cases are supported by detailed definitions in the metadata. For example, the at-times borderline distinction between *vagrant* and regular visitor to Australia was made with reference to the Convention on Migratory Species and the decisions of the BirdLife Australia’s Rarities Committee, along with definitions to deal with cases such as when a small number of individuals arrived together, bred but failed to establish a viable population. Feeding and Breeding habitats aggregate habitat types defined in a national classification system into 31 and 17 categories respectively judged relevant to birds. Notwithstanding, conversion of source data to these categories required considerable interpretation based on knowledge of habitats and Australian birds, and was undertaken by experienced senior ornithologists (mostly STG).

Body mass is a key biological attribute for which underlying source data are exceptionally disparate in quality and quantity and with varying levels of attribution to age, gender and subspecies. We have provided detailed source data (average, minimum, maximum) for males, females and unsexed birds, preferencing measurements for adults where this was available and documented, along with a field documenting our sources. These are used as a basis for calculation of three taxon summary fields (average, minimum, maximum) by methods documented in detail in our metadata (Data Citation 1, file ‘Australian_Bird_Data_Version_1_Metadata.csv’).

Many sources provide data only for species, or only patchily for subspecies, so attribution of it to subspecies is problematic and limited. We have provided data for at least some, and often all subspecies in 142 (61.7%) of fields. It is provided for most fields relating to taxonomy, status and distribution. Among biological and ecological fields, subspecies data is more limited but includes some body mass, egg size and mobility fields, and all climate metric fields, the latter based on detailed mapping of subspecies undertaken in the course of that study^14^. An absence of data is coded as NA (not assessed) or NAV (not available). All data presented for subspecies explicitly relate to that subspecies (i.e. are not simply re-iterated from the parent species).

A further and major source of intepretational challenge arises with ascribing usage as ‘non-trivial’. This was essential in order to avoid attributions based on one-off or accidental records; for example, a seabird blown inland by storm winds cannot reasonably be considered to make use of the woodland habitat in which it finds itself. Preliminary test extractions by a second person demonstrated the often highly subjective nature of ‘non-trivial’, prompting revision of the metadata to further minimise it. ‘Non-trivial’ is defined for each data field in our metadata as more than 1% of quantitative records, and in anecdotal terms as (for example) including ‘occasionally’ but not ‘very occasionally’.

Fields subject to high levels of interpretation based on ‘non-trivial’ usage and conversion of source data to defined categories are denoted in the *Source of data* field of the Metadata file with ‘interpretation this database’. It is anticipated that most users will wish to use even the fields of greatest interpretational subjectivity as these interpretations have been made systematically by expert Australian ornithologists based on documented methods and referenced sources; indeed, the systematisation of such information across all Australian taxa is a key value of this database.

## Data Records

Data, metadata and references are presented in csv files, and as an alternative the three are combined as worksheets in an Excel file (all in Data Citation 1).

Data are presented in ‘Australian_Bird_Data_Version_1.csv’ as a row for each taxon and columns for data. Data headers start with a Column number for ready matching with the Metadata and conclude with a Category number (Table 2).

Metadata, with a row of information for each field in the data file, are provided in ‘Australian_Bird_Data_Version_1_Metadata.csv’ under the following topic headings: Column number, Column header, Category number, Category, Definition, Taxa assessed, Field codes, Explanation of field factor codes, Source of data and Notes.

References are cited in the Data and Metadata files using the author-date system and listed in alphabetical order in ‘Australian_Bird_Data_Version_1_References.csv’.

The Excel file, ‘Australian_Bird_Data_Version_1.xlsx’, includes colour coding of some fields for ease of use but which do not provide additional information. It also includes formulae for calculation of taxonomic uniqueness and relative brain size which are not retained in csv files.

## Technical Validation

The database was extensively checked for structural anomalies such as inappropriate data types or field codes (all fields) and inconsistencies between related fields. We also conducted quality checks on a random sample of 100 taxa for each of seven groups of fields (Table 4). These took the form of extraction of data for these taxa from primary original sources blind to the current dataset and by a person different to the original extractor. Test extractions were compared to database entries and discrepancies attributed by a third person (the assessor, always DCF) to: a. database error; b. test error; or c. interpretational discrepancy. Because the original extractor frequently had access to additional sources of information (as documented in our Metadata), these assessments were then offered to the original extractor for comment, and some discrepancies attributed to a forth category, d. data from other sources, final decisions about which were always made by the assessor. We here report (Table 4) only finalised database errors and interpretational discrepancy rates.

Errors and interpretational discrepancies were confined to fields requiring moderate to high levels of interpretation and, in particular, decisions about triviality of use and attribution to categories where the source data did not employ categorisation or consistent categorisation across all taxa (Table 4). Some discrepancies between database and test results also arose because the database reflected information additional to that in the key source.

### Usage Notes

Issues with interpretation during data extraction are discussed above. In addition, an appreciation of the nature of systemic uncertainty is key to sound use of the data. Generically, uncertainties are either *epistemic* (associated with the state of our knowledge) or *linguistic* (associated with the terms and definitions employed)^29^. For example, body mass data reflect sampling issues including time-of-day, time-of-year, age, gender, regional variation and measurement error, all of which are epistemic uncertainties^30^. Similarly, flight-initiation distances may vary between areas with different prevailing exposure to human activity^31^. In contrast, the nature of habitat and food substrate categories is a *linguistic* problem both in definition and in imposing arbitrary boundaries on habitats that may intergrade.

Two taxonomic sorting fields are provided with subspecies in alphabetical order by scientific name. These follow alternate recent published representations of avian evolutionary relationships. The dataset may also readily be subsetted for any given analytical requirement using sort and filter options available in various database software packages. Filterable fields in the main database include, for example, Species, Core species, Extinct taxa, Vagrant taxa, Introduced taxa, Endemic taxa (either entirely or breeding only), and taxa occurring in particular sub-jurisdictions (states and territories) and territorial islands.

## Additional Information

**How to cite this article:** Garnett, S. T. *et al.* Biological, ecological, conservation and legal information for all species and subspecies of Australian bird. *Sci. Data* 2:150061 doi:10.1038/sdata.2015.61 (2015).

## Supplementary Material



## Figures and Tables

**Table 1:** Taxa presented in Australian Bird Data Version 1 (Data Citation 1)

**Taxon level**	**Core** *	**Extinct**	**Introduced**	**Vagrant**	**Total**
species	725	12	27	151	915
subspecies	931	21	20	76	1048
population†	na‡	na	na	na	58
supplementary§	na	na	na	na	35
**Total row entries**	**1656**	**33**	**47**	**227**	**2056**

*Core taxa are extant species and subspecies that occur regularly and naturally in Australia, i.e. that are not extinct, introduced or vagrant

^†^ entries are provided separately for non-breeding (visiting) populations of seabirds where the species or subspecies also breeds in Australia

^‡^ na=not applicable

^§^ erroneous records and introduced taxa that have died out.

**Table 2:** Categories of data fields provided in Australian Bird Data Version 1 (Data Citation 1)

**Field category (category no.)**	**No. of fields**	**Example field(s) (type of data)**
Sequence (1)	2	Taxon sort (no.)
Taxonomy & nomenclature (2)	23	Genus name (name), Species (1/0/NA)*, Ultrataxon uniqueness (value)
Phylogeny (3)	3	Hackett coarse clade (category)
Australian population status (4)	12	Endemic (entirely in Australia) (1/0/NA), Endemic (breeding only) (1/0/NA)
Conservation status (5)	10	Global IUCN status 2015 (category)
Legal status (6)	16	EPBC status July 2015 (national, category), CAMBA (China Australia Migratory Bird Agreement, 1/not listed)
Distribution (7)	29	New South Wales (categories), Christmas Island (categories), Also in Papua New Guinea (categories)
Morphology (8)	19	Taxon average body mass (value), Brain volume (value)
Habitat (9)	48	Feeding habitat: Terrestrial: Arid shrubland (1/0/NA), Breeding habitat: Beaches and sand cays (1/0/NA)
Food (10)	11	Food: fruit (1/0/NAV/NA), Food: fish / invertebrates (marine) (1/0/NAV/NA)
Behaviour (11)	4	Mean flight initiation distance (value)
Breeding (12)	15	Nest location: Burrow (1/0/NA), Nest aggregation: Colonial (1/0/NAV/NA)
Mobility (13)	12	Nature of international movements (category), No. of banded birds recovered (value)
Climate metrics (14)	24	[climate change] Sensitivity index (value), Climate model factor - Annual mean temperature (%)
Administration (15)	2	BirdLife Australia Taxon ID (code)
**Total fields**	**230**	

*Binary fields with ‘NA’=not applicable or not assessed; sometimes also with NAV=not available.

**Table 3:** Types of data provided in 230 fields in Australian Bird Data Version 1 (Data Citation 1)

**Field type**	**n fields**	**% of fields**
Numeric value	62	27.0
Class value	2	0.9
Ranked category	21	9.1
Binary	97	42.2
Other unranked category	25	10.9
Name	11	4.8
Notes	8	3.5
Other*	4	1.7

*taxonomic sequence numbers and administrative codes.

**Table 4:** Fields, data types, discrepancy rates and interpretational issues in quality check tests of Australian Bird Data Version 1 (Data Citation 1)

**Field category**	**Fields [description]**	**Nature of data**	**Level of interpretation**	**Test taxa**	**n fields checked**	**n species checked** *	**n cells checked**	**% error in database**	**% interpretational discrepancies**	**Source of interpretational discrepancies**
Taxonomy & nomenclature	Species, Subspecies, Ultrataxon	binary	None	all	3	100	296	0	0	
Australian population status	Population description	16 categories	Moderate	species	1	100	100	0	0	
Australian population status	Core, Non-breeding population, Extinct, Introduced, Vagrant, Supplementary	binary	Slight	species	6	95	570	0	0	
Conservation status	Global IUCN status 2014	9 categories	None	species	1	99	99	0	0	
Distribution	[Occurrence on] Continental islands and Offshore islands only	binary/tertiary	Slight	all	3	95	285	0	0	
Habitat	[breeding habitats]	binary	Great	species	17	87	1479	1.1	2.1	misalignment of habitat categories between database and sources, and failure of source to discriminate between breeding and other habitat
Breeding	Nest aggregation: Solitary, Colonial, Parasitic	binary	Moderate	species	3	78	234	0.6	1.9	borderline cases between solitary and dispersed (loose) colonies

*this was often less than the 100 taxa randomly selected because the test extractor proved unable to identify suitable data from the key source.
